# *Bacillus siamensis* Improves the Immune Status and Intestinal Health of Weaned Piglets by Improving Their Intestinal Microbiota

**DOI:** 10.3390/microorganisms12051012

**Published:** 2024-05-17

**Authors:** Huawei Liu, Xinyu Liu, Haiyang Liu, Jiaqi Tang, Wei He, Tianqi Xu, Baojing Cheng, Baoming Shi, Jianchun Han

**Affiliations:** 1 College of Animal Science and Technology, Northeast Agricultural University, Harbin 150030, China; s210501032@neau.edu.cn (H.L.); xinyuliu0212@163.com (X.L.);; 2 National Soybean Engineering Technology Research Center, Heilongjiang Academy of Green Food Science, Northeast Agricultural University, Harbin 150028, China

**Keywords:** *Bacillus siamensis*, weaned piglet, microbiota, short-chain fatty acid, immunity, gut permeability

## Abstract

Previous studies on the early interference of gut microbiota by *Bacillus siamensis* (*B. siamensis*) in weaned piglets are rarely reported, and the present trial is a preliminary study. This experiment was conducted to investigate the effects of *B. siamensis* supplementation on the growth performance, serum biochemistry, immune response, fecal short-chain fatty acids and microbiota of weaned piglets. Sixty weaned piglets were randomly divided into a control group (CON) and a *B. siamensis* group (BS), which were fed a basal diet and the basal diet supplemented with 5 × 10^10^ CFU *B. siamensis* per kg, respectively. Each group had 3 replicates and 10 piglets per replicate. The trial lasted for 28 days. The results showed that *B. siamensis* significantly increased the serum growth hormone (GH) and insulin-like growth factor (IGF) in piglets. Compared with the CON group, the levels of serum immunoglobulin and inflammatory factors in the BS group were significantly improved. In addition, the serum concentrations of zonulin and endotoxin (ET) in the BS group were lower. The dietary addition of *B. siamensis* significantly increased fecal short-chain fatty acid (SCFA) levels in piglets. Notably, *B. siamensis* improved the microbial composition by increasing beneficial genera, including *Weissella*, *Lachnospiraceae_NK4A136_group* and *Bifidobacterium*, and decreasing pathogenic genera, including *Pantoea*, *Fusobacterium* and *Gemella*, in piglet feces. Correlation analysis showed that the benefits of dietary *B. siamensis* supplementation were closely related to its improved microbial composition. In summary, the addition of *B. siamensis* can improve the immunity function, inflammatory response, gut permeability and SCFA levels of weaned piglets, which may be achieved through the improvement of their microbiota.

## 1. Introduction

Weaning is a stressful phase of the pig growth cycle and often has a negative impact on the growth performance and gut health of piglets. Especially with the early weaning strategies implemented in modern farming, piglets are challenged by environmental, dietary and psychological emergencies that can lead to immune dysfunction, impaired gut barrier function and microbiota homeostasis, resulting in impaired growth and even death [1]. In recent decades, antibiotics have been widely used as feed additives to reduce the damage caused by weaning stress [2]. Antibiotics, as growth promoters, improve feed utilization and reduce bacterial pathogen infections, largely improving economic returns for producers [3]. However, antibiotics have the potential to jeopardize human health by increasing the tolerance of pathogenic bacteria and residues in livestock products [4]. Reducing the use of antibiotics is an inevitable trend, and since 2006, the European Union and many countries around the world have officially banned the use of antibiotics in feed [5]. Therefore, finding safe and effective antibiotic-replacement strategies is important for the healthy and sustainable development of the pig industry.

Probiotics are known to be an effective and safe alternative to antibiotics [6]. The composition and metabolic activity of the gut microbiota co-evolves with the host from the time it leaves the mother’s body and is influenced by the host’s diet and lifestyle [7]. Gut microbes interact with the host and influence the host’s physiology, nutrition and health [8]. Furthermore, gut microbes can positively influence the host’s immune response, anti-inflammatory capacity and gut barrier through the production of metabolites and short-chain fatty acids (SCFAs) [9]. Therefore, the early intervention of the gut microbiota is an effective way to relieve weaning stress. Studies have shown that probiotics can improve the composition of the gut microbiota, increase colon SCFA levels and promote growth in weaned piglets [10]. Recent research evidence suggests that probiotics can alter the gut microbiota and alleviate systemic inflammatory responses [11]. Therefore, we hypothesize that probiotics can effectively alleviate piglet weaning stress by intervening in the composition of the gut microbiota at an early stage, which ultimately positively affects the growth and health status of piglets.

Given the direct and indirect contribution of probiotics to piglet health, their potential to optimize piglet health is currently being investigated [12]. Probiotics, such as *Bacillus subtilis*, *Lactobacillus plantarum* and *Clostridium butyricum*, have been shown to have a positive effect on growth performance, immune levels, the integrity of gut barrier function and the gut microbiota in weaned piglets [13,14,15]. *Bacillus siamensis* (*B. siamensis*) is a kind of probiotic with good resistance to acids, alkalis, bile salt and high temperatures. However, there are limited studies on the effects of *B. siamensis* on the health of weaned piglets. Therefore, the aim of this study was to investigate the effects of the supplementation of diets with *B. siamensis* on the growth performance, immunity level, gut permeability, and fecal SCFAs and microbiota of weaned piglets.

## 2. Material and Methods

The Northeast Agricultural University Ethical Committee for Animal Experiments (Grant Number: NEAU-[2013]-9) approved all animal experiments conducted at the Bayan Kangrun Husbandry Co. (Harbin, China).

### 2.1. Animals and Diets

Sixty crossbred castrated male and female (Duroc × Large White × Landrace) weaned piglets (30 days of age) with an initial body weight (BW) of 8.63 ± 0.1 kg were randomly divided into control and experimental groups according to sex and BW. Piglets were weaned on the evening of the 30th day after birth. There were 3 replicates per treatment group and 10 pigs per replicate. The control group (CON) was fed a basal diet, and the *B. siamensis* group (BS) was fed the basal diet supplemented with *B. siamensis*. The experimental diet was prepared by mixing the basal diet with *B. siamensis* bacterial solution at a concentration of 5 × 10^10^ CFU/kg. This concentration is based on previous experimental studies conducted by the laboratory team. The *B. siamensis* strain was stored in the Chinese Typical Culture Preservation Center (CCTCC NO: M 202297). The piglet basal diets were formulated to meet or exceed the nutritional requirements recommended by the National Research Council (NRC, 2012) [16]. The trial period lasted for 28 days from early on the 31st day to the evening of the 58th day after the birth of piglets. All weaned piglets in this trial were used for this study only. The ingredients and chemical composition of the basal diets are detailed in Table 1. All piglets were housed in pens (2.5 m × 4.0 m) in which the temperature was maintained at 26–28 °C and the relative humidity at 55–65%. All piglets were allowed to feed and drink at will during the experimental period.

### 2.2. Sample and Data Collection

The feed intake of the piglets was recorded once a week, and the fasting BW of piglets was recorded on days 1 and 28 to determine the average daily gain (ADG), average daily feed intake (ADFI) and feed conversion ratio (FCR) of piglets. On day 28 of the experiment, two piglets per replicate close to the mean BW were selected and blood was obtained by puncturing their anterior vena cava with a sterile needle. The blood samples were then centrifuged at 4 °C for 10 min at 3000× *g* to obtain the supernatant serum, which was stored at −80 °C for the determination of serum biochemical, immunological, inflammatory, gut permeability and growth hormone-related parameters. Fresh feces from piglets (the same batch of pigs from which serum was collected) were collected by massaging the rectum and stored at −80 °C for the analysis of microbial community and SCFAs.

### 2.3. Determination of Serum Biochemistry

Serum biochemical markers included Urea nitrogen (UN), total protein (TP), albumin (ALB), globulin (GLB), urea nitrogen (UN), creatinine (CREA), aspartate transaminase (AST), alanine aminotransferase (ALT), alkaline phosphatase (ALP), total cholesterol (TCHO), triglyceride (TG), glucose (GLU), high-density lipoprotein cholesterol (HDL) and low-density lipoprotein cholesterol (LDL). Analyses were performed using an Automatic Biochemical Analyzer (7160 autoanalyzer; Hitachi, Tokyo, Japan). The kit was purchased from the Nanjing Institute of Construction Bioengineering (Nanjing, China), and the trial steps were performed following the manufacturer’s notes.

### 2.4. Determination of Immunity, Inflammation, Gut Permeability and Growth Factors

The levels of immunoglobulin A (IgA), immunoglobulin G (IgG), immunoglobulin M (IgM), interleukin-6 (IL-6), interleukin-10 (IL-10), tumor necrosis factor-alpha (TNF-α), diamine oxidase (DAO), D-lactic acid (D-LA), endotoxin (ET), zonulin, growth hormone (GH) and insulin-like growth factor (IGF) were measured using Enzyme-Linked Immunosorbent Assay (ELISA) kits from Shanghai Enzyme-linked Biotechnology Co., Ltd. (Shanghai, China). The experimental procedures were performed in strict accordance with the manufacturer’s instructions.

### 2.5. Analysis of SCFAs

SCFA analysis of piglet fecal samples was performed using a GC-FID system (model 7890A; Agilent Technologies, Santa Clara, CA, USA) equipped with an AOC-20s autosampler, a capillary column (HP-INNOWAX (19091N-133), 30 m × 0.53 mm × 1.0 µm) and a flame ionization detector. Specifically, 2 g of piglet feces was macerated in 2 mL of ultrapure water in a freezer at 4 °C for 48 h. The feces were then centrifuged at 10,000 rpm/min for 10 min at 4 °C, and this step was repeated twice. Filtration of the supernatant was carried out using a 0.22 μm filter membrane. Finally, the filtrate was combined with an internal standard solution (25% metaphosphate solution containing crotonic acid) at a volume ratio of 5:1. The injector temperature, detector temperature and oven temperature were set to 230 °C, 240 °C and 180 °C, respectively, and the pressure was set to 90kPa. The flow rates of nitrogen, air and hydrogen were 20, 400 and 30 mL/min, respectively.

### 2.6. DNA Extraction, PCR and Library Construction and Sequencing

Total genomic DNA was extracted from the piglet feces sample by the CTAB/SDS method. DNA concentration and purity were monitored on 1% agarose gel. Primes 341F (5′-CCTAYGGGRBGCASCAG-3′) and 805R (5′-GGACTACNNGGGTATCTAAT-3′) were used to construct PCR amplification for the V3-V4 region of the bacterial 16S rDNA gene. The amplified products were purified by using a gel extraction kit (Darmstadt, Germany). Purified PCR products were evaluated using an Agilent 2100 Bioanalyzer (Agilent, Santa Clara, CA, USA) and Illumina’s (Kapa Biosciences, Woburn, MA, USA) library quantification kit with qualified library concentrations above 2 nM. Finally, the libraries were sequenced on an Illumina NovaSeq platform to generate 250 bp paired-end reads.

For the double-ended data obtained by sequencing, the sample needed to be data-split according to barcode information, and the joint and barcode sequences were removed. Then, the data were spliced and filtered, and then, DADA2 was invoked with qiime DADA2 denoise-paired for length filtering and denoising. The Amplicon Sequence Variant (ASV) feature sequence and an ASV abundance table were obtained, and singleton ASVs were removed. The SILVA and NCBI databases were used to annotate species according to the ASV sequence files, and the abundance of species of each level in each sample was counted according to the ASV abundance table. The diversity index was calculated using the R language vegan package (V.2.5–7), and the rank sum was used to test the significance of the differences between the two groups. The Mann–Whitney U test was used to screen phylum- and genus-level differential species.

### 2.7. Analysis of Data

All data were statistically analyzed by IBM SPSS 26.0 statistics using a two-tailed unpaired Student’s *t*-test. Data are expressed as mean ± SEM. *p* < 0.05 indicates statistically significant differences.

## 3. Results

### 3.1. Growth Performance

The growth performance results are summarized in Table 2. Compared with the CON group, dietary *B. siamensis* supplementation had no significant effects (*p* > 0.05) on the initial weight, final weight, ADG, ADFI and FCR of piglets.

### 3.2. Serum Biochemistry

As shown in Table 3, the serum levels of TCHO (*p* < 0.001) and HDL (*p* = 0.001) in the BS group were significantly reduced. Moreover, the levels of UN, CREA, GLU, AST, ALT, TP, ALB, GLB, TG and LDL were not significantly affected (*p* > 0.05).

### 3.3. Serum Growth Factor

**Figure 1:**
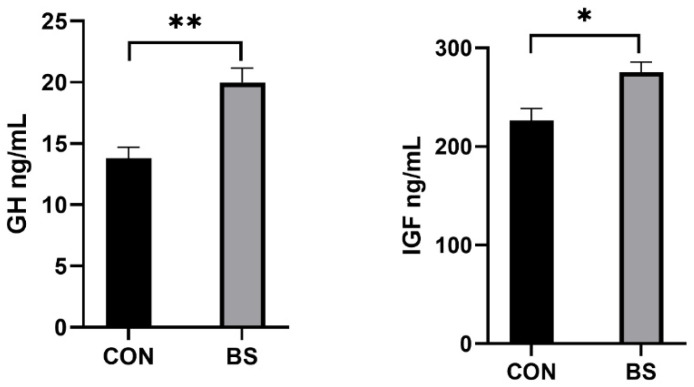
Effects of supplemental *B. siamensis* on serum growth hormone levels of weaned piglets. Data are expressed as mean ± SEM (n = 6). * *p* < 0.05, ** *p* < 0.01. CON: control; BS: *B. siamensis*.

As shown in Figure 1, the concentrations of GH (*p* < 0.01) and IGF (*p* < 0.05) in the serum of piglets in the BS group were significantly higher than in the CON group.

### 3.4. Serum Immunity and Inflammation

**Figure 2:**
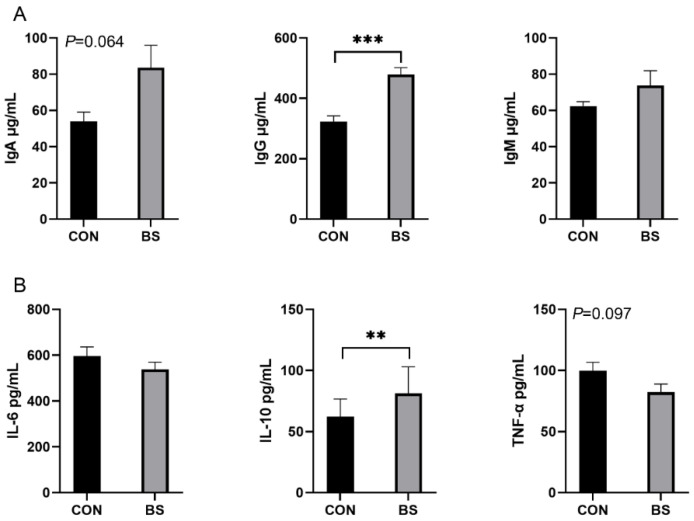
Effects of *B. siamensis* supplementation on serum immunity (**A**) and inflammation (**B**) levels of weaned piglets. Data are expressed as mean ± SEM (n = 6). ** *p* < 0.01, *** *p* < 0.001. CON: control; BS: *B*. *siamensis*.

The effects of *B. siamensis* on the serum immune and inflammatory levels of piglets are shown in Figure 2. Compared with the CON group, the serum levels of IgG were significantly higher (*p* < 0.001) in piglets in the BS group, and there was a tendency (*p* = 0.064) for IgA to be elevated, with no significant difference (*p* > 0.05) in IgM (Figure 2A). Compared with the CON group, the serum levels of IL-10 were significantly higher (*p* < 0.01) in piglets in the BS group, and there was a tendency (*p* = 0.097) for TNF-α to be decreased, with no significant difference (*p* > 0.05) in IL-6 (Figure 2B).

### 3.5. Gut Permeability

**Figure 3:**
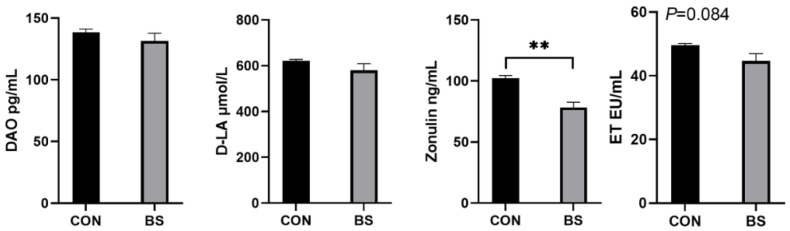
Effects of *B. siamensis* supplementation on serum levels of intestinal-permeability markers in weaned piglets. Data are expressed as mean ± SEM (n = 6). ** *p* < 0.01. CON: control; BS: *B. siamensis*.

As illustrated in Figure 3, the serum zonulin levels of piglets in the BS group were significantly decreased (*p* < 0.01), ET had a tendency (*p* = 0.084) to be decreased, and DAO and D-LA were not significantly affected (*p* > 0.05).

### 3.6. Fecal SCFA Content

**Figure 4:**
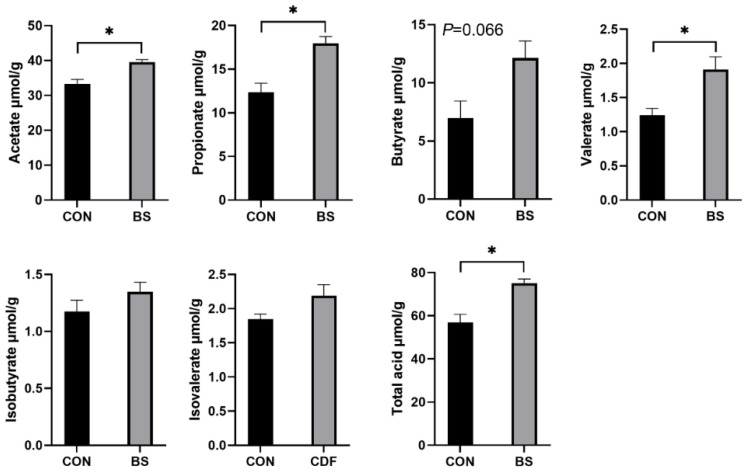
Effects of *B. siamensis* supplementation on fecal SCFA levels of weaned piglets. Data are expressed as mean ± SEM (n = 6). * *p* < 0.05. CON: control; BS: *B. siamensis*.

The effects of *B. siamensis* on the fecal SCFA content of piglets are shown in Figure 4. Dietary *B. siamensis* supplementation significantly increased (*p* < 0.05) the contents of acetate, propionate, valerate and total acid in piglets. Compared with the control group, butyrate in the BS group had a tendency (*p* = 0.066) to increase. In addition, isobutyrate and isovalerate did not differ significantly (*p* > 0.05) between the two groups.

### 3.7. Fecal Microbiota

**Figure 5:**
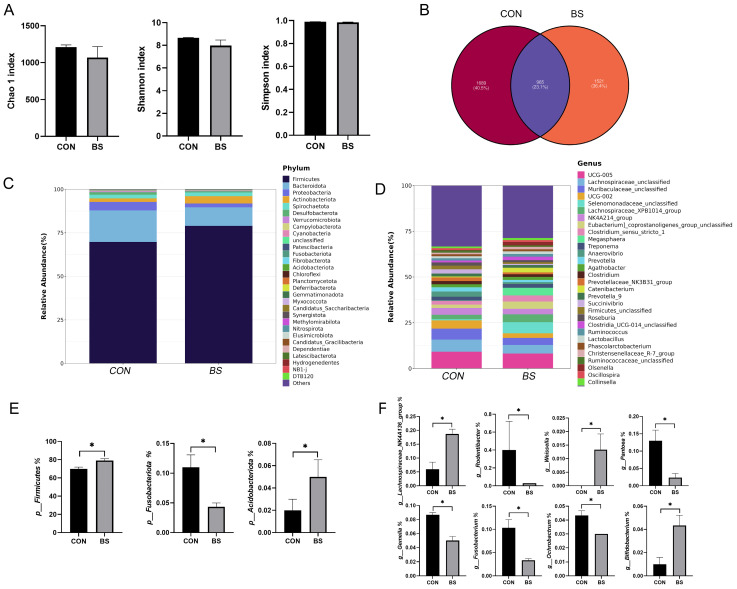
Effects of *B. siamensis* supplementation on fecal microbiota composition of weaned piglets. Comparison of the observed Chao 1, Shannon and Simpson indices (**A**), and Venn diagram of shared and specific ASVs in the fecal microbiota between CON and BS groups (**B**). Relative abundance of fecal microbiota at the phylum (**C**,**E**) and genus (**D**,**F**) levels. Data are expressed as mean ± SEM (n = 3). * *p* < 0.05. CON: control; BS: *B. siamensis*.

To investigate the effects of *B. siamensis* addition on the fecal microbiota of piglets, we sequenced the 16SrRNA gene in the fecal samples. The result is shown in Figure 5. Compared with the control group, there were no significant changes in the Chao 1, Shannon and Simpson indexes in the BS group (Figure 5A). This showed that *B. siamensis* supplementation did not affect fecal microbiota diversity. By observing the Venn diagram, it was found that there were 965 shared ASVs in both groups and 1689 and 1521 specific ASVs in the CON and BS groups, respectively (Figure 5B). At the phylum level, *Firmicutes* and *Bacteroidetes* were the dominant phyla, accounting for more than 85% of the totality (Figure 5C). At the genus level, the dominant genera were *UCG-005*, *Lachnospiraceae_unclassified*, *Muribaculaceae_unclassified*, *UCG-002*, *Selenomonadaceae_unclassified*, *Lachnospiraceae_XPB1014_group* and *NK4A214_group* (Figure 5D). Subsequently, the difference in microbial composition between the two treatments was further analyzed. At the phylum level, the abundance of *Firmicutes* and *Acidobacteriota* was significantly increased (*p* < 0.05) in the BS group and the abundance of *Fusobacteriota* was significantly reduced (*p* < 0.05) compared with the CON group (Figure 5E). At the genus level, *B. siamensis* supplementation significantly increased (*p* < 0.05) the abundance of *Weissella*, *Lachnospiraceae_NK4A136_group* and *Bifidobacterium*. Meanwhile, the abundance of *Rodentibacter*, *Pantoea*, *Gemella*, *Fusobacterium* and *Ochrobactrum* was significantly reduced (*p* < 0.05) (Figure 5F).

### 3.8. Spearman’s Correlation Analysis

**Figure 6:**
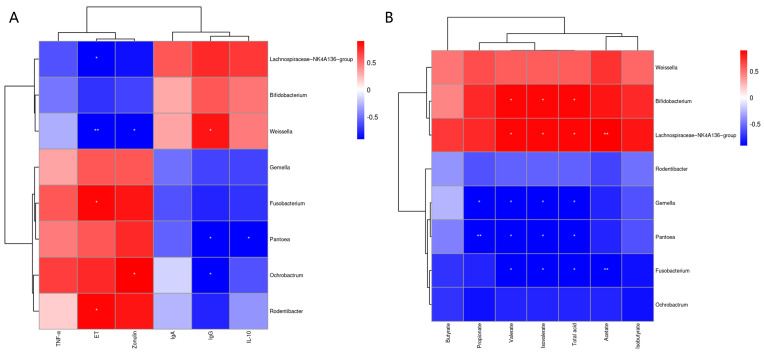
The Pearson correlation analysis of fecal microbiota with serum immune and inflammatory factors and intestinal-permeability markers (**A**). Spearman correlation analysis of fecal microbiota with fecal SCFAs (**B**). The red and blue indicate positive and negative correlations, respectively: * *p* < 0.05, ** *p* < 0.01.

The Pearson correlation analysis of fecal microbiota with serum immune and inflammatory factors and gut permeability markers is shown in Figure 6A. The results showed that serum ET was positively correlated (*p* < 0.05) with *Rodentibacter* and *Fusobacterium*, and negatively correlated with *Weissella* (*p* < 0.01) and *Lachnospiraceae_NK4A136_group* (*p* < 0.05). The serum zonulin levels were positively correlated (*p* < 0.05) with *Ochrobactrum* and negatively correlated (*p* < 0.05) with *Weissella*. The serum IgG levels were positively correlated (*p* < 0.05) with *Weissella* and negatively correlated (*p* < 0.05) with *Ochrobactrum* and *Pantoea*. There was a significant negative correlation (*p* < 0.05) between serum IL-10 and *Pantoea*. Pearson correlation analysis of fecal microbiota and fecal SCFAs is shown in Figure 6B. The results showed that Propionate was significantly negatively correlated with *Pantoea* (*p* < 0.01) and *Gemella* (*p* < 0.05). The levels of fecal valerate, isovalerate and total acid were significantly negatively correlated (*p* < 0.05) with *Fusobacterium*, *Pantoea* and *Gemella*, and significantly positively correlated (*p* < 0.05) with *Lachnospiraceae_NK4A136_group* and *Bifidobacterium*. The level of acetate in stool was negatively correlated (*p* < 0.01) with that of *Fusobacterium*, and significantly positively correlated (*p* < 0.01) with *Lachnospiraceae_NK4A136_group*.

## 4. Discussion

Piglets are challenged by dietary and environmental stresses and harmful bacteria during the weaning period, which often causes dysfunctions of the gut and immune systems and disturbances in the gut microbial ecology, ultimately negatively affecting the growth performance and health status of the piglets [17,18]. Therefore, effective mitigation or elimination of the stress associated with weaning is necessary. Probiotics are widely used in animal feed due to their advantages of regulating the host immune system, maintaining the balance of microbiota and inhibiting the proliferation of pathogens in the gut [19,20]. Therefore, this study investigated the relieving effect of *B. siamensis* on the weaning stress of piglets.

The growth performance of piglets is often limited by weaning stress [21]. Many studies have previously shown that probiotic supplementation at the weaning stage improves piglet growth performance [22,23]. However, the results of this study showed that *B. siamensis* had no significant effect on the growth performance of piglets. This is consistent with the results of Cui et al. [24]. These differences in results may be explained by inconsistencies in diet composition, piglet strain and dosage. Duddeck et al. supplemented the diet of their experimental group with 1.875 × 10^5^ CFU/g and 1.875 × 10^6^ CFU/g *B. subtilis*, respectively, and the test results showed that the ADG of piglets in the lower-dose probiotic group was significantly higher than that in the control group and the higher-dose probiotic group [25]. Nevertheless, in order to more deeply explore the effect of *B. siamensis* on piglet growth, we measured serum GH and IGF levels. The results showed that the addition of *B. siamensis* to the diet significantly increased the serum GH and IGF levels in piglets. Meanwhile, the GH/IGF axis has been shown to be a master regulator for stimulating the growth of animal cells and somatic cells [26]. In addition, the higher levels of serum GH and IGF in the BS group indicated that piglets had higher anabolic activity, which had a positive effect on body growth and development [27]. Studies have shown that high-protein diets significantly increase growth hormone and IGF levels [28]. Therefore, the upregulation of growth hormone and IGF secretion may be closely related to the fact that *B. siamensis* improved the composition of the intestinal microbiota in piglets and thus increased nutrient intake [29].

The concentration of blood biochemical parameters is a standard index to evaluate nutrient metabolism and function [30]. Serum TCHO concentration can be used as a marker of lipid metabolism [31]. In this study, we found that the serum TCHO levels of piglets supplemented with *B. siamensis* were significantly reduced. This decrease in serum TCHO concentration reflected the improvement in lipid metabolism [32]. The reason may be that the supplementation of probiotics can effectively reduce serum cholesterol levels by increasing bile salt synthesis and bile acidolysis binding [33]. It is very interesting that the results of this trial showed that the serum HDL level was significantly reduced in the BS group.

Immunoglobulins are secreted by b-cell-activated plasma cells and play an important role in humoral immunity [34]. The main functions of immunoglobulins include activation of the complement system, which is responsible for inhibiting the attachment of microbiota, and inhibiting bacterial metabolism by blocking enzymes, antimicrobials and neutralizing viruses, thereby preventing pathogenic effects [35]. In this study, we showed that the addition of *B. siamensis* to the diet increased IgA and IgG levels in weaned piglets. This result is consistent with that reported by Zong et al. [36]. In addition, in the present study we found that *B. siamensis* supplementation increased IL-10 and decreased TNF-α. IL-10, as a master anti-inflammatory regulator with multiple functions, can antagonize pro-inflammatory factors and inhibit the migration of inflammatory cells [37]. Meanwhile, TNF-α is an inflammation-initiating factor that can initiate TNFR2 signaling through the activation of TNFR1, causing an inflammatory response in the body [38]. Overall, *B. siamensis* alleviated the inflammatory response and improved immunity levels in piglets.

A well-functioning gut barrier effectively prevents pathogens, toxins and antigens from entering the circulation through the gut mucosa [39]. Therefore, we measured the levels of four serum gut-permeability-related biomarkers (DAO, D-LA, ET and zonulin). Although the differences in serum DAO and D-LA were not significant, this study showed that serum zonulin and ET concentrations were reduced in piglets fed *B. siamensis*. The levels of gap connexin zonulin and endotoxin were significantly reduced, indicating reduced gut permeability [40,41]. It has also been previously reported that probiotics play a positive role in maintaining the integrity of gut epithelial cells and improving gut permeability [42]. This may be attributed to the fact that *B. siamensis* regulates the composition of the gut microbiota and promotes the secretion of SCFAs.

SCFAs, as end products of gut microbial fermentation [43], have been shown to have a variety of beneficial effects on immunity and metabolism [44]. Meanwhile, previous studies have shown that the supplementation of probiotics can increase the content of gut SCFAs in piglets, thereby alleviating the negative effects of weaning stress [45]. Similarly, our study showed that *B. siamensis* significantly increased SCFAs in piglet feces. Furthermore, SCFAs have been shown to be involved in various physiological processes and have positive effects on host health and nutrition [46]. Butyric acid, in particular, is thought to be a source of metabolic energy for gut cells and has anti-inflammatory properties that help the host maintain healthy gut barrier function [47]. Thus, the increased concentration of SCFAs may be one of the reasons why immunity, inflammation and gut permeability are improved in *B. siamensis*-fed piglets.

The gastrointestinal microbiota is critical for the metabolism of nutrients, the growth and maturation of immune responses and protection against pathogens [48]. The gut microbiota is a dynamic ecosystem that is influenced by a variety of factors, including diet, lifestyle, age and genotype. Diet is particularly important [49]. In this study, we investigated the effects of the addition of *B. siamensis* on microbial composition at the phylum and genus levels. At the phylum level, the abundance of *Firmicutes* in the feces of piglets from the BS group was significantly higher than that of the CON group, while the abundance of *Bacteroidetes* was lower, suggesting that the ratio of *Firmicutes* to *Bacteroidetes* was increased in the CON group. It has been reported that the increased ratio of *Firmicutes* to *Bacteroidetes* may be related to increased energy production and nutrient intake [50,51]. This may be due to improved nutrient utilization and absorption by dietary *B. siamensis* supplementation. In addition, the increased abundance of *Firmicutes* contributes to the synthesis of butyrate and propionate [52]. At the genus level, we found that the abundance of *Weissella*, *Lachnospiraceae_NK4A136_group* and *Bifidobacterium* in the BS group was significantly higher than that in the CON group. *Weissella* belongs to the facultative anaerobic lactic acid bacteria that can control foodborne pathogens by producing bacteriocins and organic acids [53]. *Lachnospiraceae_NK4A136_group* has been suggested to have multiple benefits, including the improvement of gut permeability and the production of SCFAs [54,55]. In addition, it has been shown that *Lachnospiraceae_NK4A136_group* is negatively correlated with inflammation [56]. *Bifidobacterium*, as a typical probiotic, can produce key anti-inflammatory SCFA propionate and inhibit the reproduction of harmful bacteria [57,58]. In addition, this study also found that the abundance of *Pantoea*, *Fusobacterium* and *Gemella* was significantly reduced. *Pantoea* is a Gram-negative bacterium that can cause associated infections that harm host health [59]. *Fusobacterium* is well known as an aggressive and pro-inflammatory disease-causing bacterium [60,61]. *Gemella* is a catalase-negative, Gram-positive bacterium and a potential pathogenic agent in the gastrointestinal tract [62,63]. According to this, dietary *B. siamensis* can improve the gut microbial composition of piglets by increasing the abundance of beneficial bacteria and inhibiting the abundance of pathogenic bacteria.

Many studies have shown a strong link between gut microbiota and host health [64,65]. Spillman correlation analysis showed that harmful bacteria (*Rodentibacter*, *Pantoea*, *Fusobacterium* and *Ochrobactrum*) with significantly lower abundance were negatively correlated with host immune levels, anti-inflammatory factors and SCFAs, and positively correlated with gut permeability markers. The increased abundance of beneficial bacteria (*Weissella* and *Lachnospiraceae_NK4A136_group*) was positively correlated with immune levels and SCFAs, and negatively correlated with gut permeability markers. Previous studies have shown that the gut microbiota has many benefits for the host, including activating the immune system, producing beneficial metabolites in gut fermentation, and preventing the proliferation of pathogenic bacteria [66]. In addition, SCFAs are the main source of energy for gut epithelial cells and are known for their antioxidant and anti-inflammatory properties [67]. Overall, *B. siamensis* improved immunity, inflammation, gut permeability and SCFAs in piglets by increasing the abundance of beneficial bacteria and decreasing the abundance of harmful bacteria.

## 5. Conclusions

Our results suggest that the supplementation of *B. siamensis* in the diet improves growth hormones, immunity, inflammatory factors, gut permeability and SCFAs in weaned piglets, and that all of these benefits are closely linked to the positive modulation of the microbiota by *B. siamensis*. More importantly, this study provides a new potential nutritional strategy for the alleviation of weaning stress in piglets. Meanwhile, this study also has certain limitations, which require experiments under different conditions to increase the sample size, such as different concentrations of probiotics or pigs with different growth periods. In addition, it is necessary to optimize the administration form of probiotics and determine the exact administration amount in test piglets to further verify the effectiveness of *B. siamensis*.

## Figures and Tables

**Table 1:** Dietary ingredients and nutrient contents of the basal diets (from 30 to 58 days of age).

Items	Content
Ingredient amount (%)	
Extruded corn	49.05
Soybean meal	3.50
Extruded soybean	12.50
Yeast culture bio-yeasture	2.00
Fish meal	2.00
Mung bean pulp protein powder	1.25
Flour	20.00
Soybean phospholipid powder	1.25
Beer yeast powder	0.50
Whey powder	1.25
Limestone	0.40
Glucose	1.25
Salt	0.35
Choline chloride	0.40
Mildew preventive	0.15
Vitamin ^1^ mix	3.00
Mineral ^2^ mix	1.15
Total	100
Nutrition level (calculated value ^2^)	
Digestible energy (Mcal/kg)	3.49
Crude protein (%)	16.85
Calcium (%)	0.58
Total phosphorus (%)	0.47
L-lysine (%)	0.96
L-methionine (%)	0.39
L-methionine + L-cysteine, (%)	0.61
L-threonine (%)	0.65
L-tryptophane (%)	0.19

^1^ Supplied per kg diet: 10,500 IU Vitamin A, 2500 IU Vitamin D_3_, 83.33 mg Vitamin E, 5.00 mg Vitamin K_3_, 2.67 mg Vitamin B_1_, 10.00 mg Vitamin B_2_, 6.00 mg Vitamin B_6_, 0.05 mg Vitamin B_12_, 48.00 mg niacinamide, 24.00 mg pantothenic acid, 1.60 mg folic acid, 0.25 mg biotin. ^2^ Supplied per kg diet: 79.50 mg iron, 7.50 mg copper, 60.00 mg zinc, 19.50 mg manganese, 0.50 mg iodine, 0.35 mg selenium.

**Table 2:** Effects of *B. siamensis* supplementation on growth performance of weaned piglets.

Items	CON ^1^	BS ^1^	SEM ^2^	*p*-Value
Initial weight (kg)	8.63	8.66	0.10	0.817
Final weight (kg)	17.51	17.94	0.69	0.565
ADG ^1^ (g/d)	316.99	331.48	23.68	0.574
ADFI ^1^ (g/d)	473.50	467.70	18.05	0.764
FCR ^1^	1.50	1.42	0.07	0.287

^1^ CON: control; BS: *B. siamensis*; ADG: average daily gain; ADFI: average daily feed intake; FCR: feed conversion ratio. ^2^ Data are expressed as mean ± SEM; data are mean values of 3 replicates per treatment (10 piglets per replicate).

**Table 3:** Effects of *B. siamensis* supplementation on serum biochemical parameters of weaned piglets.

Items	CON ^1^	BS ^1^	SEM ^2^	*p*-Value
UN ^1^, mmol/L	4.71	3.35	0.85	0.167
CREA ^1^, mmol/L	77.62	69.57	8.58	0.380
GLU ^1^, mmol/L	6.36	5.55	0.58	0.202
AST ^1^, IU/L	88.98	103.30	10.85	0.263
ALT ^1^, IU/L	100.07	108.50	12.91	0.528
ALP ^1^, IU/L	382.40	300.80	56.61	0.180
TP ^1^, g/L	48.84	46.40	1.79	0.205
ALB ^1^, g/L	25.88	24.57	1.65	0.444
GLB ^1^, g/L	22.96	21.84	0.75	0.169
TG ^1^, mmol/L	0.82	0.62	0.09	0.114
TCHO ^1^, mmol/L	2.72 ^a^	2.10 ^b^	0.62	<0.001
HDL ^1^; mmol/L	1.11 ^a^	0.77 ^b^	0.34	0.001
LDL ^1^; mmol/L	1.27	1.20	0.12	0.563

^a,b^ Means with different superscripts in each row are statistically different (*p* < 0.05). ^1^ CON: control; BS: *B. siamensis*; UN: urea nitrogen; CREA: creatinine; GLU: glucose; AST: aspartate aminotransferase; ALT: alanine aminotransferase; ALP: alkaline phosphatase; TP: total protein; ALB: albumin; GLB: globulin; TG: triglyceride; TCHO: total cholesterol; HDL: high-density lipoprotein cholesterol; LDL: low-density lipoprotein cholesterol. ^2^ Data are expressed as mean ± SEM (n = 6).

## Data Availability

The data are contained within the article.
